# Mechanism of Fish Habitat Selection Based on Hydrodynamic Indicators Under Multiple Topographic Conditions for Ecological Restoration Requirements

**DOI:** 10.1002/ece3.71933

**Published:** 2025-09-10

**Authors:** Han Liu, Guosheng Yang, Junqiang Lin, Dongsheng Wang, Wei Xu, Hao Jiang, Di Zhang, Lingquan Dai, Wei Jiang, Sha Li

**Affiliations:** ^1^ Yangtze Eco‐environment Engineering Research Center China Three Gorges Corporation Wuhan Hubei China; ^2^ National Engineering Research Centerof Eco‐environment Protection for Yangtze River Economic Belt China Three Gorges Corporation Wuhan Hubei China; ^3^ Institute of Hydroecology, MWR&CAS Wuhan Hubei China; ^4^ State Key Laboratory of Simulation and Regulation of Water Cycle in River Basin China Institute of Water Resources and Hydropower Research Beijing China; ^5^ China Renewable Energy Engineering Institute Beijing China

**Keywords:** CART methodology, fish behavior, habitat restoration, hydrodynamic indicator, suitability index

## Abstract

The establishment of hydropower projects has altered the flow regime and structure of fish habitats. Various habitat restoration measures based on natural river morphology have been implemented, but the effects of habitat restoration and the hydrodynamic mechanisms of fish preferred topography remain unclear. We constructed diverse near‐natural microtopographic habitats and monitored test fish behavior in real time using a Radio Frequency Identification (RFID) system with Passive Integrated Transponder (PIT) tags. Results demonstrate that structurally distinct microtopographies differentially attract fish. Among four selected hydrodynamic indicators, flow velocity and vorticity most strongly influenced *Onychostoma sima* ( *O. sima* ). The optimal vorticity for fish habitats ranges between 1.75 and 10.85/s, with suitable flow velocities exceeding 0.545 m/s. Using Habitat Suitability Index (HSI) and Classification and Regression Tree (CART) methodologies, we revealed the hydrodynamic mechanism underlying habitat selection. Validation via the Receiver Operating Characteristic (ROC) method confirmed that the hydrodynamic‐based habitat selection model effectively predicted suitable habitats for most test fish. This study elucidates linkages between fish activity and hydrodynamic indicators in complex terrain, identifying habitat utilization patterns under variable flow and topographic conditions. Our findings provide theoretical support for ecological operation and habitat management in hydropower‐affected rivers.

## Graphical abstract

Fish habitats are often characterized by diverse topographic conditions, which, under the influence of varying flow rates, create a wide range of flow patterns. As fish are direct perceivers of water flow, changes in hydrodynamic conditions significantly impact their habitat selection behavior. To quantify the mechanisms through which hydrodynamic conditions influence fish behavior, we conducted real‐time monitoring and statistical analysis of the time fish spent in different regions under various conditions. By correlating these observations with hydrodynamic indicators of the corresponding flow rates, we identified flow velocity and vorticity as the most critical factors guiding fish habitat preferences. The study revealed that fish exhibit distinct habitat preferences under different flow conditions, suggesting the need to design suitable flow conditions in diverse topographic settings based on threshold values of preferred hydrodynamic indicators.

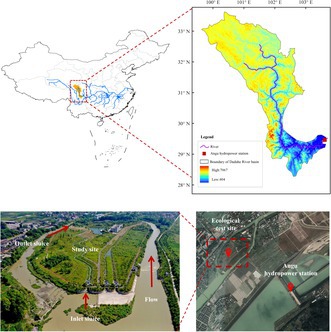

## 1 Introduction

The establishment of hydropower projects has significant comprehensive benefits such as flood control, power generation, and shipping, but on the other hand, they have affected the flow regime and structure of fish habitats (Wang et al. 2019). The construction of hydropower projects has altered the natural environment of river ecosystems, leading to changes in the location and structure of habitats for many freshwater species (Barbarossa et al. 2020). River ecological restoration techniques are effective measures to improve habitat and biodiversity (Li et al. 2022), and the study of the effects of hydrological alterations caused by hydropower projects in combination with fish passage barriers, geomorphological changes, and other contemporaneous stressors is the current focus of fish habitat restoration (Hecht et al. 2019).

The physical structure of rivers is intricately linked to the diversity of fish habitat communities. In natural river systems, rivers predominantly exhibit meandering morphology, forming a variety of geomorphic units such as main channels, tributaries, riverine wetlands, pool‐riffle sequences, and so on (Wang et al. 2019). The most widely used foreign classification of river types is the one proposed by Leopold and Wolman in 1995, which classifies rivers into straight, meandering, and braided based on plane configuration (Leopold and Wolman 1957). In addition, the classification of rivers into three categories of curved forms, waved forms, and central bars based on the difference of river planform considering hydrological and sediment characteristics is also widely accepted. The straight river is a single river channel with a straight plane shape and uniform flow pattern; the bent rivers have a unique characteristic of a spiral flow field, often forming a bend loop, which is the result of the combined effect of centrifugal force and water pressure; braided and networked rivers are mostly due to the dense sandbars in the river channel, and the sandbars block the flow to divert and form a confluence at the end of the sandbars, resulting in a significant oblique flow effect. Studies have shown that fish prefer to inhabit and breed in river flow environments with special morphology. Deep pools and shallows are common habitat types for fish, and changes in flow magnitude can cause inversions in the flow velocities of deep pools and shallows (Thompson 2011).

The behavior of fish exhibits variability in response to nonuniform flow, including variations in velocity and turbulence. Hydraulic models can provide detailed water flow information on a large spatial scale, and three‐dimensional models are more accurate in describing the spatial distribution of flow characteristics or the magnitude of variables compared to two‐dimensional models. Traditional hydraulic studies have focused on parameters such as flow velocity and water depth, which are inadequate for fully characterizing or qualitatively describing the spatial flow characteristics of complex river reaches (Crowder and Diplas 2002). Therefore, many scholars have carried out studies on hydrodynamic conditions such as vorticity, turbulent kinetic energy, and shear stress of different river structures (Shamloo et al. 2001; Yagci 2010). Based on this, research combining complex hydraulic conditions with the behavioral patterns of fish has also been widely conducted. For example, in indoor flumes, the addition of deflectors to alter the flow field (velocity, turbulence kinetic energy) and bed morphology revealed that fish prefer to move and rest in diversified habitats shaped by deflectors (Wang et al. 2021). By adding cylindrical structures to indoor flumes, cylindrical vortex structures were found to be important for fish holding stations and swimming stability, and Karman gaits and spills were significantly affected by velocity, vorticity, and Reynolds shear stress (Zha et al. 2021).

A significant amount of ecological restoration of river habitats has been carried out worldwide, with restoration of physical river structures being a primary approach (Griffith and McManus 2020). Based on the results of the abovementioned laboratory test results, restoration activities at small to medium scales include improvements in riverbed substrates, rehabilitation of bank structures, construction of small weirs and dams, placement of large wood materials, and large‐scale interventions such as dam removal or modification (Lei et al. 2023). However, considering the growth cycle of fish and external disturbance factors, the effect evaluation of fish habitat restoration projects must be assessed on different temporal and spatial scales. The lack of systematic evaluation and inconsistent assessment approaches contribute to the limited scientific evidence regarding the success of river restoration efforts (Kail et al. 2015; Wyzga et al. 2021). Large‐scale restoration projects are more likely to achieve success, but they often face constraints due to available resources and conflicting interests (Lake et al. 2007). On the other hand, small‐ to medium‐scale restoration efforts can address specific issues through the integration of eco‐hydraulics and fish behavior studies. From the perspective of hydrology, fish life histories and population dynamics are influenced by flow regimes (Koehn et al. 2019). From the perspective of river structures, artificial or seminatural structures have been implemented in many large dammed river systems (Staentzel et al. 2020). From the perspective of fish resources, the assessment of river restoration efforts often focuses on changes in fish populations (Marttila et al. 2019). In summary, quantitative measurement studies of the relationship between channel morphology, flow conditions, and habitat use of fish populations are scientific tools that can improve the effects of habitat restoration on small and medium scales and provide a theoretical basis for large‐scale restoration.

To investigate the habitat preference of fish under complex flow conditions, clarify the habitat selection mechanism of fish under various terrain conditions, and provide a theoretical basis that can be directly applied to practice for the habitat restoration project of river physical structure, in this study, we adopt the passive integrated transponder (PIT) radio frequency identification system to monitor the habitat behavior of fish in the self‐constructed area modeled after the natural river in the Angu ecological test site. Combined with the three‐dimensional hydrodynamic simulation of different flow rates in the test river, we explored the habitat selection mechanism of different fish species under the complex hydrodynamic flow field of various microtopography.

## 2 Materials and Methods

### 2.1 Study Site

**FIGURE 1:**
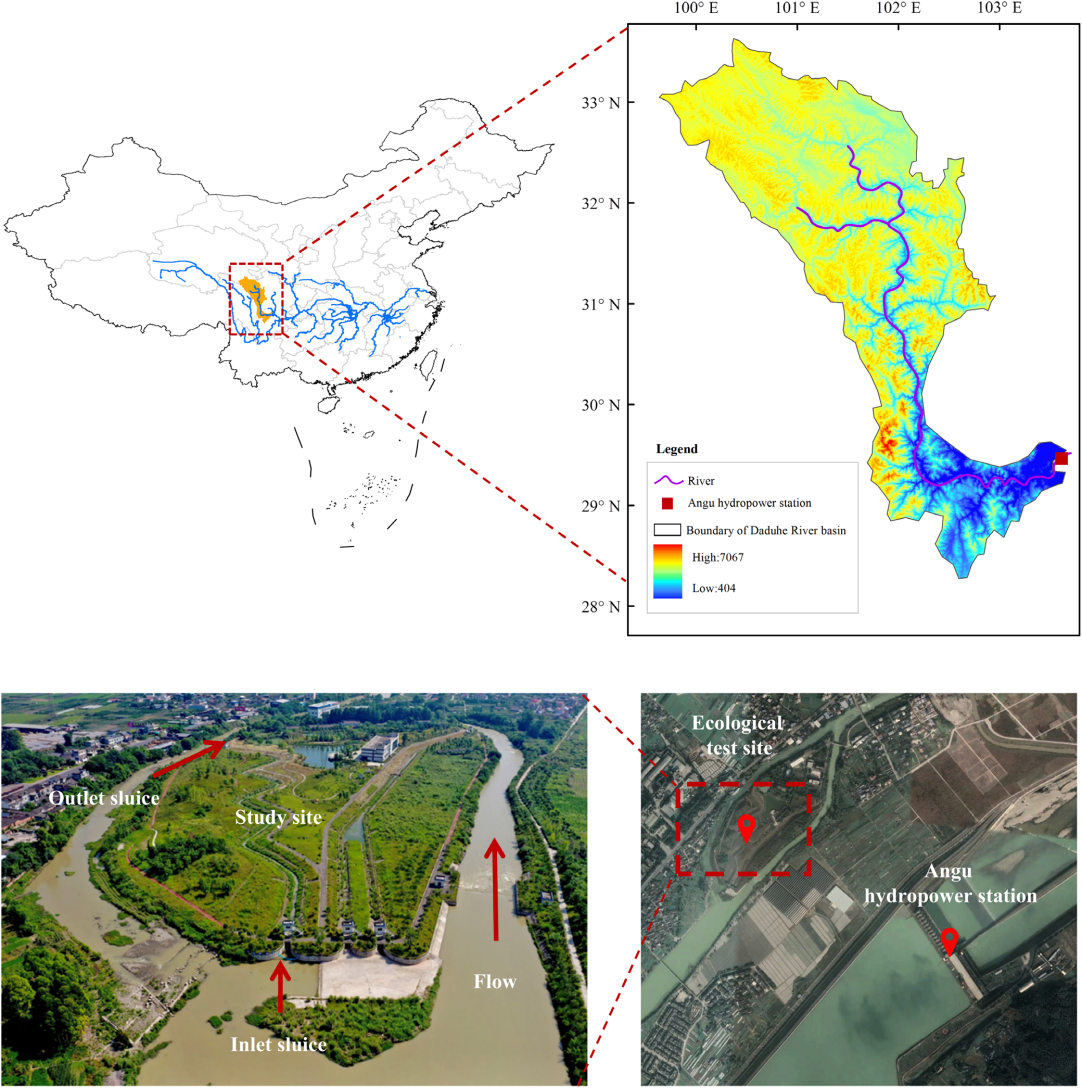
Location diagram of the Angu Ecological Test Site.

Angu Hydropower Station is in Leshan City, Sichuan Province, China, which is a typical large ecological test site for ecological restoration theory and technology research of river network (Figure 1). The river channel used in this study is a meandering river channel in the Angu ecological test site, and the slope and substrate materials are laid out according to the ecosystem environment of the local river network area; the typical vegetation of the local riverbank grows naturally on the slope. The experimental channel is designed with a maximum water depth of 2.0 m. Under the various flow conditions tested, water depths range between 0.7 and 1.5 m, with no significant differences. The channel bed is constructed with the following layers, from bottom to top: a 20 cm thick layer of screened soil, a geocomposite membrane, a 15 cm thick combined layer of coarse sand cushion and gravel cushion, and a 25 cm thick layer of gabion baskets for impermeability and channel stabilization. The surface test layer consists of small‐to‐medium sized gravel, with a particle size range of 30–50 mm, selected based on the habitat preferences of the target fish species. The design of the morphological parameters of the bend section is based on the empirical relationship equation proposed by Soar (Soar 2000). In the actual construction process, there are slight differences in the description parameters of the bend sections.

### 2.2 Research Reach Setup

To weaken the influence of anomalous flow patterns in the upstream and downstream inlet and outlet sluices, a meandering river section of approximately 140 m in the middle was selected as the test channel. Based on remote sensing imagery and actual surveys, the Angu River network area is characterized by numerous sandbars and a dense distribution of deep pools and shallow riffles. In the test channel, Channel 1 was set up, where two groups of pool‐riffles of different sizes were constructed manually (Pool 1: 0.3 m below the riverbed, Riffle 1: 0.5 m above the riverbed; Pool 2: 0.4 m below the riverbed, Riffle 2: 0.7 m above the riverbed). Channel 2 was set up, and two groups of sandbars of different sizes were built manually, with reference to the distribution of parameters of the actual riverbed planform (Bar1: bottom length 2.4 m, bottom width 0.9 m, height 1.3 m; Bar2: bottom length 4 m, bottom width 1.5 m, height 1.5 m).

### 2.3 The Measurement of Terrain and Flow Fields

We used the i70II inertial navigation version of the Pocket RTK system for terrain measurement in a designated river segment within the test area. The measured parameters included latitude, longitude, and elevation. The planar accuracy of the measurements was ±1.2 mm, while the elevation accuracy was ±2 mm. In total, 3000 measurement points were selected for river channel modeling and flow field simulation.

Additionally, an Acoustic Doppler Velocimeter (ADV) was used to measure the three‐dimensional surface velocity data for selected flow conditions within the actual measurement test area. These data were used to adjust the model parameters. Three actual velocity measurement sections were set up at the interface between straight and curved river segments, the entrance of a shallow shoal, and the exit of a sandbar. Four flow conditions were measured: 0.3, 0.6, 1, and 1.5 m^3^/s. Due to the difficulty of measurement, two other flow conditions were not measured.

### 2.4 Test Fish Treatment

#### 2.4.1 Test Fish Species

According to the fish resource survey results conducted around the Angu Ecological Test Site, the target fish species for fish passage at the Angu Hydropower Station and the main protected fish species in the Angu region, the test fish selected was *Onychostoma sima*. This species predominantly occupies river reaches characterized by high‐velocity, turbulent flows and gravel‐stone substrates and exhibits benthic swimming and foraging behaviors. For this study, 30 individuals of the test fish were selected from the breeding station at the experimental field and surrounding fish farms. The selected fish exhibited good vitality and similar sizes (Table 1). The study was conducted from August 7, 2022, to September 15, 2022.

**TABLE 1:** Parameters of morphological characteristics of test fish.

Species	Age/year	Total length/mm		Body length/mm		Body weight/g	
		Range	Average	Range	Average	Range	Average
*Onychostoma sima*	1–2	200.0–260.0	234.0	175.0–230.0	189.7	76.5–172.4	113.7

#### 2.4.2 Fish Maintenance

In this study, the test fish were temporarily reared in a circular open pond at the fish breeding station of Angu Hydropower Station, 100 m away from the test river, with water from the Dadu River at a temperature of 12°C ± 1°C and the dissolved oxygen level maintained at more than 6 mg/L. All of them were kept under active conditions.

#### 2.4.3 Fish Tracking

The RFID system based on PIT technology is an effective method for monitoring fish behavior in complex river environments. The unique identification code of each PIT tag is recorded when the fish bearing it enters the magnetic field range of the antenna connected to the transceiver. After briefly anesthetizing the test fish using MS‐222, the PIT tags (2.12 × 12 mm, 0.1 g, 134.2 kHz) were implanted into the dorsal muscle of the fish. Subsequently, the anesthetized fish were placed back into clear water without anesthesia, and they typically regained consciousness and resumed normal swimming behavior within 1 to 2 min. The test fish were then kept in the holding tank for an additional 2 weeks. During this period, none of the test fish died, and the wounds in their dorsal muscles healed significantly.

### 2.5 Fish Behavior Monitoring

**FIGURE 2:**
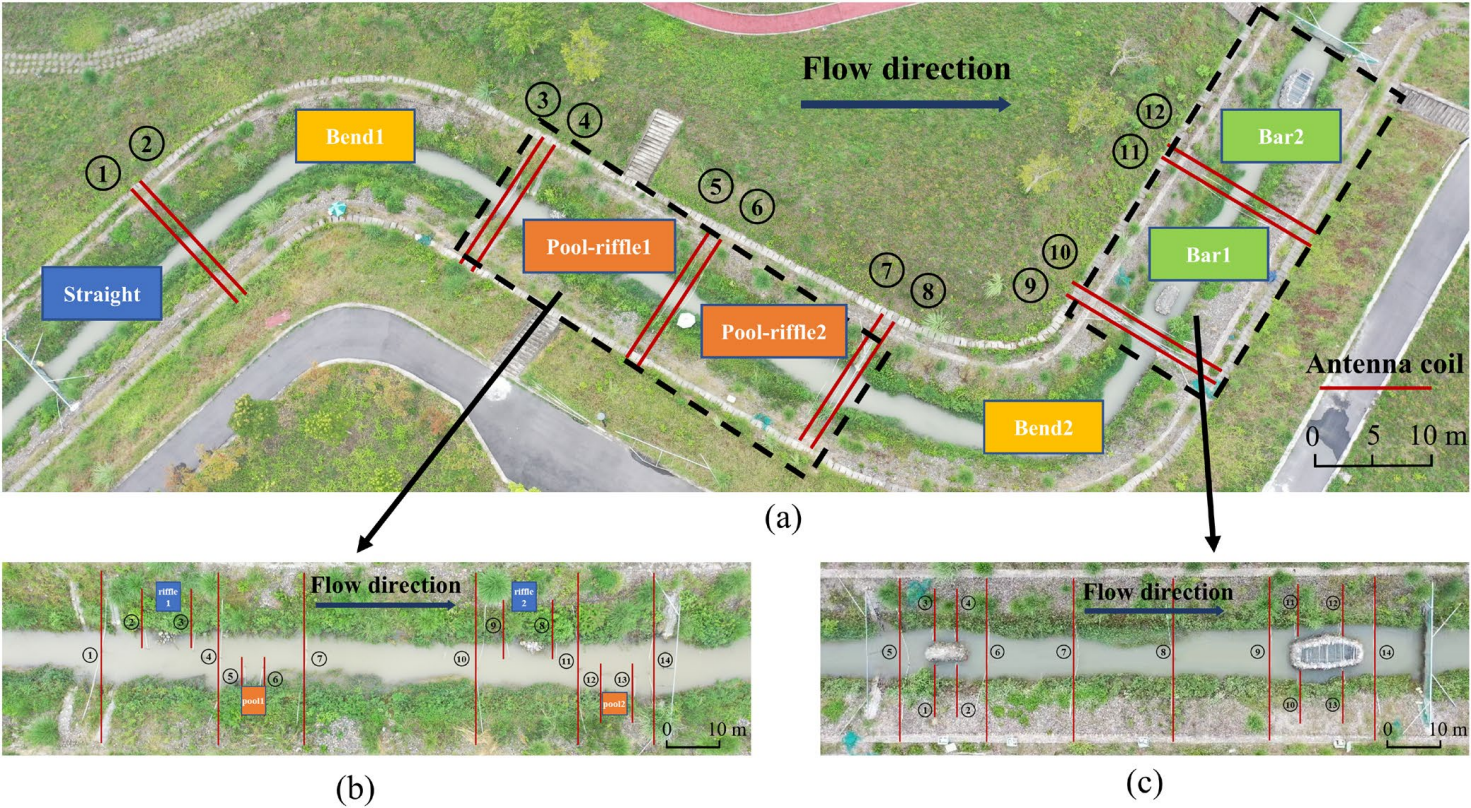
Distribution setting of the test area. Multiterrain combination test area (a). Pool‐riffle terrain test area (b). Sandbar terrain test area (c). The red line represents the monitoring coil.

All test fish were placed in the test river during the experimental period. The flow rate in the river was maintained at a low level of 0.1 to 0.3 m^3^/s for a continuous period of 7 days to allow the test fish to regain their natural swimming abilities. After sufficient acclimation, three groups of fish habitat selection tests were conducted by changing the opening of the inlet gate to alter the flow rate in the river. The upper and lower limits of the flow conditions were designed based on the velocity range of different terrains (0.2 to 2.8 m/s) under natural ecoflow conditions in the Angu River network area. Multiple groups of radio frequency identification systems (RFIDs) were deployed in the river to divide the test area and monitor fish behavior. Considering the signal interference of multiple devices over short distances and the limitation of the number of devices, the experiment was divided into three groups as follows (Figure 2).

The experiment was set up with six different flow conditions (0.3, 0.6, 1, 1.5, 2, and 3 m^3^/s), with each condition lasting for 9 h. The residence time of the test fish under different flow conditions in the straight river segment, two sets of curved river segments, two sets of pool‐riffle segments, and two sets of sandbar segments was determined by analyzing the frequency and timing of the signals obtained from coils placed at various cross‐sections (Figure 2a). After the overall experiment was completed, considering the geomorphological complexity of the sandbar and pool‐riffle segments, 14 sets of coils were installed in each of these two segments to monitor the residence time of the experimental fish in different areas within the two segments (Figure 2b,c).

### 2.6 Analysis Methods

#### 2.6.1 PIT Tag Information Statistics

The metrics obtained from PIT signals include the frequency of fish passing through a specific area and the duration of fish occupancy in each monitoring zone. The frequency statistics require filtering the signal reception count of each test fish in each coil in HyperTerminal. The calculation of occupancy duration follows a custom rule for fish habitat time, counted by the time interval between fish passing through the front and rear coils, implemented using Python 3.0.

#### 2.6.2 Flow Field Data Simulation

In accordance with Section 2.3, the terrain model was used to perform grid partitioning and flow field simulation using Fluent (Ansys 2021R1). The model was divided into 856,077 tetrahedral meshes with an average cell quality of 0.83, an average skewness of 0.236, and an average aspect ratio of 1.853, which met the mesh quality requirements. The steady‐state continuity equation and Reynolds‐averaged Navier–Stokes (RANS) equations were solved using the finite volume method, along with the closure equations for the standard k‐ε turbulence model. Due to the varying shape of the experimental channel, the pressure interpolation during equation discretization was performed using the PRESTO scheme, the volume ratio was calculated using the second‐order windward format, and the pressure–velocity coupling was handled using the coupled algorithm. Convergence was considered achieved when the residuals were below 0.0001 or when the residuals showed little change with each iteration. The inlet boundary condition was specified as a velocity inlet, with flow velocities ranging from 0.6 to 1.2 m/s based on measured values for each condition. The outlet boundary condition was set as outflow. The roughness coefficient was set to 0.035 according to the substrate characteristics. The error rate between the measured values at 16 sampling points on the surface of the three monitoring cross‐sections and the simulated values was 90.96%.

#### 2.6.3 Fish Habitat Suitability Calculation

Due to the limitation of the number of radio frequency devices, it is not possible to monitor the habitat time of fish in other terrains while monitoring the pool‐riffle/sandbar section in detail. Therefore, based on the 9 h long‐duration monitoring results in multiterrain combination test, we derived the habitat time ratios of fish in straight sections (1 area) and two groups of curved sections (2 areas) with pool‐riffle sections and sandbar sections under each flow condition and estimated the habitat time distribution of test fish in 21 areas under six flow conditions, that is, the habitat time distribution of test fish in a total of 126 flow fields with different flow conditions. To obtain the habitat suitability of fish in various flow conditions, the proportion of time fish spend in different areas to the total time should be calculated. This proportion should then be multiplied by the reciprocal of the area of the respective region to obtain the preference index of fish for that specific area. Finally, normalization of these indices will yield the habitat suitability of the flow field.
(1)

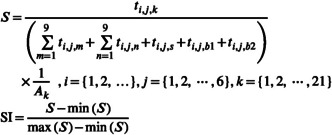

where *i* refers to the species of test fish, *j* refers to 6 different flow conditions, *k* refers to 21 different river regions, *t*_*i,j,m*_ refers to the average habitat time of 9 regions in the pool‐riffle section, *t*_*i,j,n*_ refers to the average habitat time of 9 regions in the sandbar section, *t*_*i,j,s*_ refers to the average habitat time in the straight section, *t*_*i,j,b1*_ and *t*_*i,j,b2*_ refer to the average habitat time in the curved sections, *A*_*k*_ refers to the area of 21 river regions, and SI refers to the normalized habitat suitability index.

#### 2.6.4 Algorithm for Habitat Selection

The habitat selection mechanism of fish is determined using the classification and regression tree (CART) methodology. In this method, a binary recursive partitioning technique is implemented, and the Gini coefficient is used as the metric for feature splitting. The sample is divided into two subsets, creating two branches at each nonleaf node of the decision tree. The CART method is suitable not only for binary attributes but also for continuous attributes. Even when there is autocorrelation among the feature indicators, CART can still be used to identify and predict complex hierarchical relationships among multivariate data.

The data samples are divided into a training set and a validation set in a 7:3 ratio. After the construction of the classification tree, the reliability of the predictions on the validation set is assessed using the receiver operating characteristic (ROC) method. The ROC method is widely used internationally as an effective tool for establishing “binary classification” discrimination models, as it does not depend on the scale of the test results.

## 3 Results

### 3.1 Hydrodynamics

**FIGURE 3:**
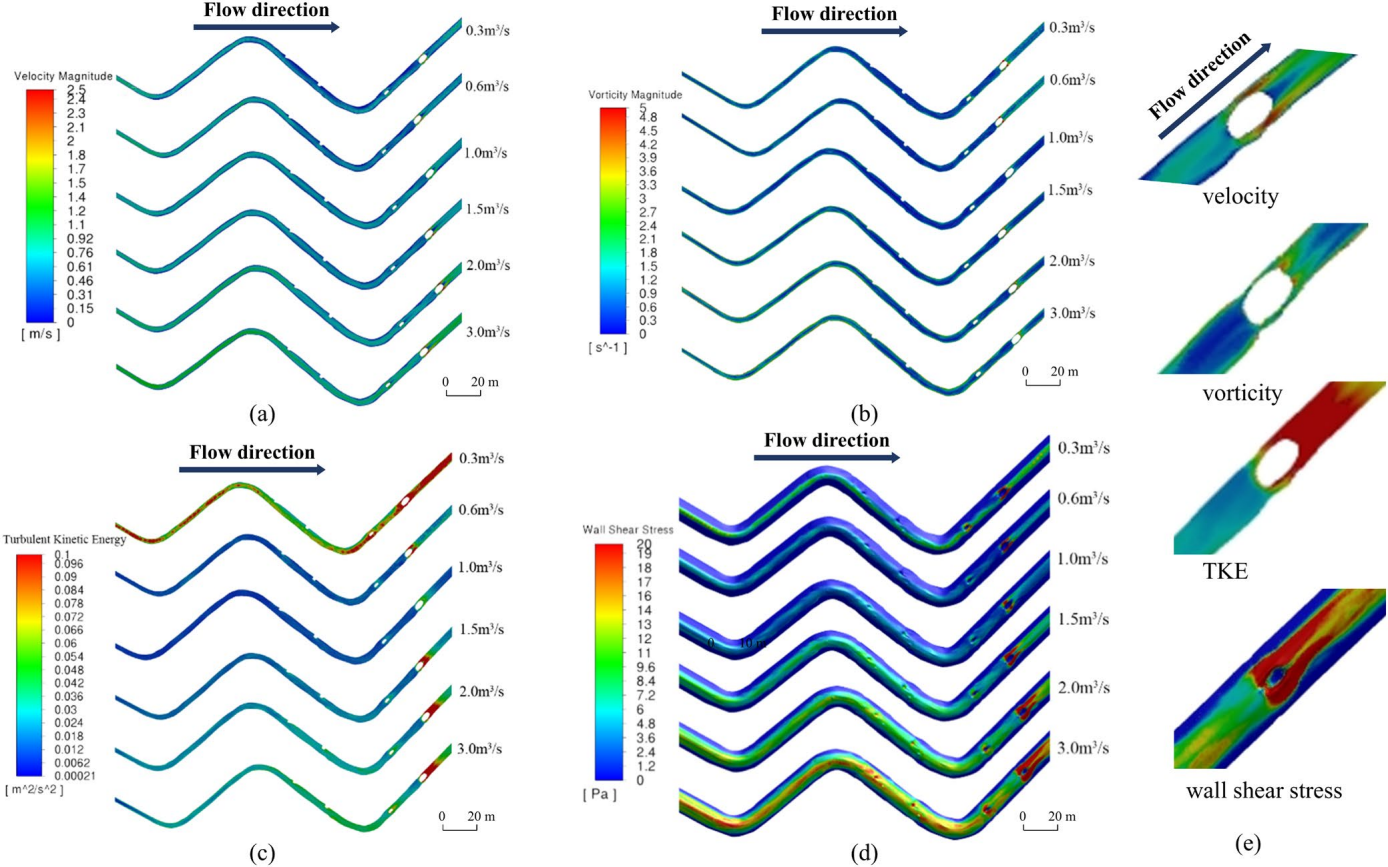
Flow field simulation results. Velocity distribution (a). Vorticity distribution (b). Turbulent kinetic energy distribution (c). Bed shear stress (d). Localized hydrodynamic variations in the sandbar segment under a 3 m^3^/s flow condition (e).

The flow velocity, vorticity, turbulent kinetic energy, and wall shear stress were selected as hydrodynamic simulation indexes to refer to the speed of water flow, the intensity of vortex motion, the degree of water turbulence, and habitat stability (Figure 3). Since the test fish are benthic fish, they are mostly active in the lower and middle layers of the test channel, so the lower section of the channel was selected in the flow field simulation results. This section was parallel to the bottom of the riverbed, 30 cm away from the bottom of the riverbed, and crosses the monitoring antenna coil. The simulation results showed that the four hydrodynamic indexes exhibited a smooth upward trend in each river section under the four working conditions with flow rates of 1.0 and 3.0 m^3^/s. At flow rates of 0.3 and 0.6 m^3^/s, the velocity and turbulence of the water flow were higher than those at higher flow conditions (1 m^3^/s) due to the low flow rate, low water depth, and simulation section close to the surface water, especially the increase in turbulent kinetic energy throughout the 0.3 m^3^/s working condition. This phenomenon primarily arises from the substantial geometric variations of the local riverbed and banks (Crowder and Diplas 2000; Lane et al. 1994). In shaping microtopography, the eddy volume generally increases in the bend of the curved river section; the deep pools and shallows have an impact on the local hydrodynamic conditions, mainly in the bed shear stress and the changes of the flow velocity in the surface layer of the shallows and the deep pools, and the sandbar section has a significant surge of all hydrodynamic indicators in the sandbar section due to the barrier effect of the sandbar. Each hydrodynamic factor shows different distribution patterns in the test channel under different flow conditions.

### 3.2 Fish Behavior

**FIGURE 4:**
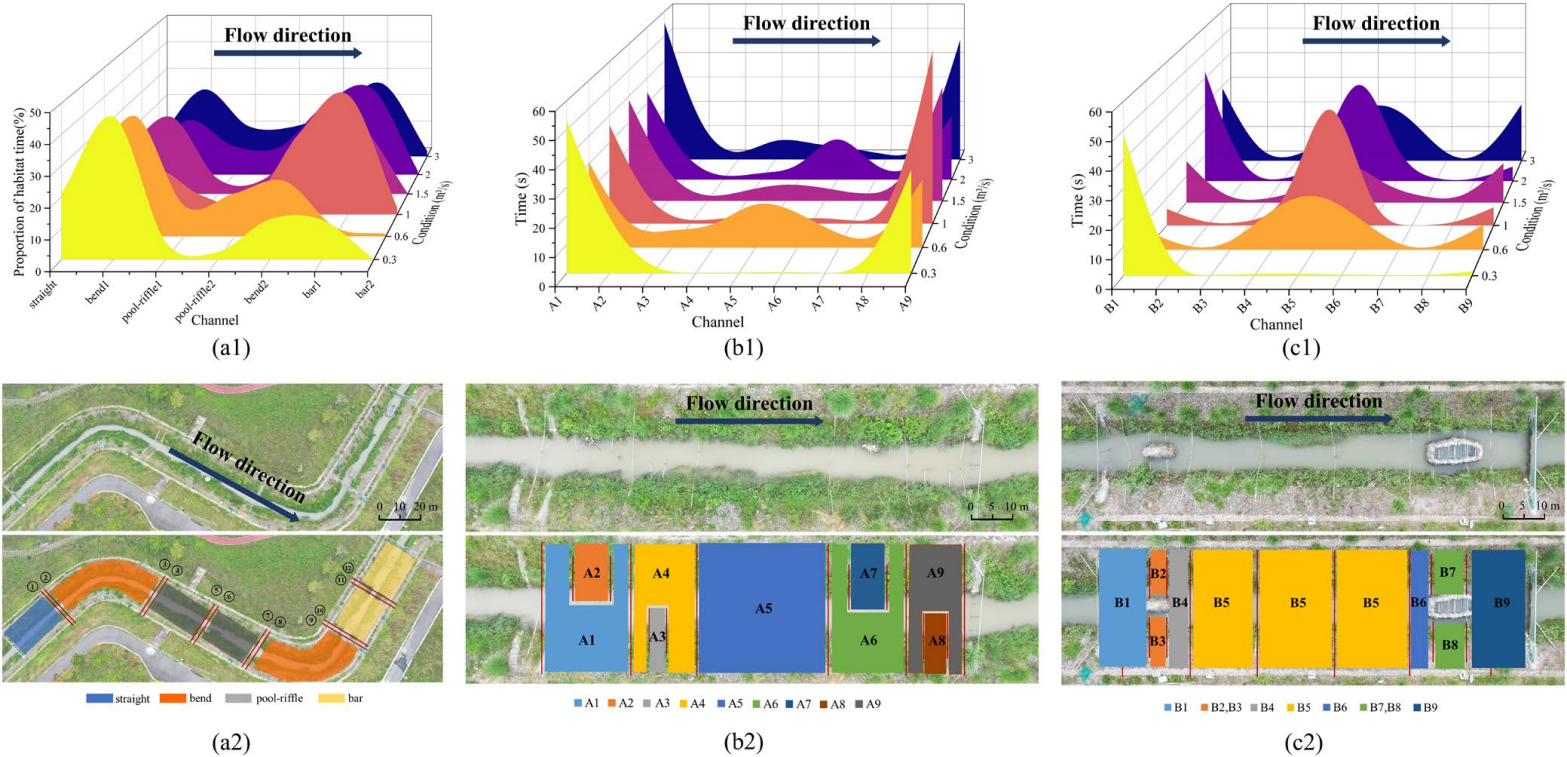
Average habitat time per fish for the test fish in each test area (a1). Zonal control of the test river section (a2). Average habitat time of test fish in each area of pool‐riffle reach (b1). Monitoring area distribution of pool‐riffle reach (b2). Average habitat time of test fish in each area of the sandbar reach (c1). Monitoring area distribution of the sandbar reach (c2).

In the multi‐terrain combination test, analysis of the average per‐fish residence time of the test fish species in each test area showed that (Figure 4) microtopography exerts varying levels of attraction to the test fish under different flow conditions. *O. sima* preferred to inhabit straight and curved river segments when the flow rate was below 0.6 m^3^/s. However, as the flow rate exceeded 0.6 m^3^/s, the fish's preferred habitat gradually shifted towards pool‐riffle and sandbar segments. In the pool‐riffle segments, the test fish spent over 60% of their time in areas A1 and A9, which are located near the shallow shoals, across all flow conditions. In the sandbar segments, the fish showed a strong preference for areas B1, B5, and B9, all of which are located at the inlets and outlets of the sandbars, spending over 90% of their time in these regions.

### 3.3 Fish Habitat Suitability

**FIGURE 5:**
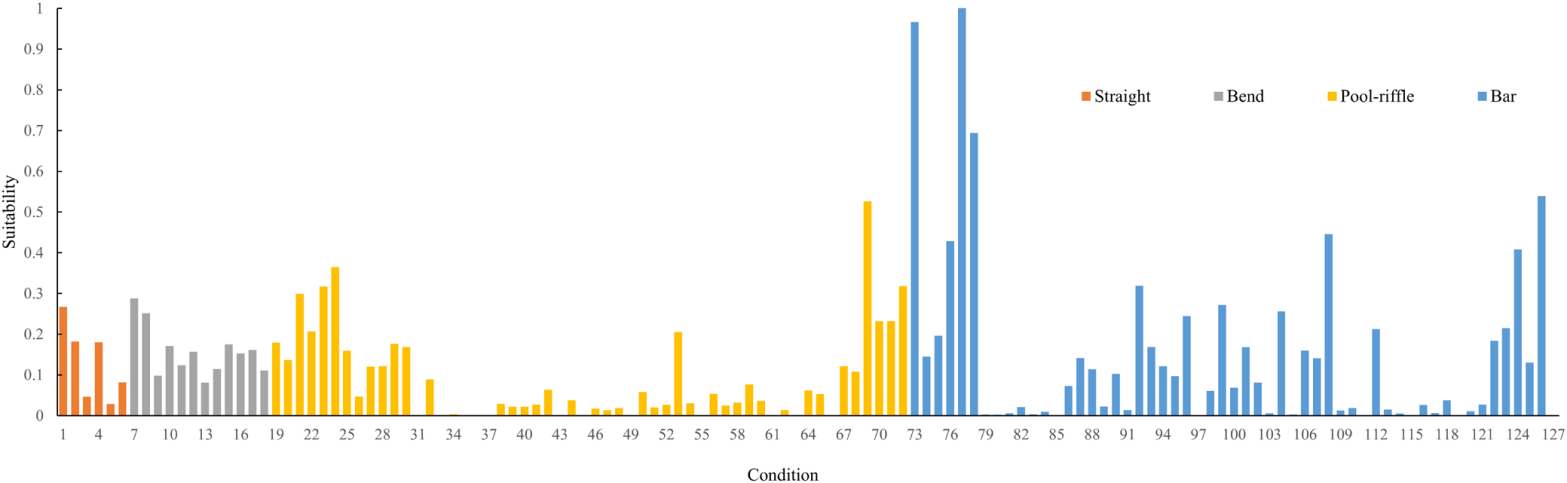
Habitat suitability distribution of test fish.

Fish habitat suitability was calculated based on the average habitat time monitored in the 21 monitoring areas under 6 flow conditions as described in Equation (1), with test fish corresponding to 126 data sets (Figure 5).

### 3.4 Fish Habitat Selection Mechanisms

There was a complex coupling relationship between the hydrodynamic indicators that affected each other. Accordingly, we further explored the combination relationship between different hydrodynamic indicators and the combination relationship between different flows.

**FIGURE 6:**
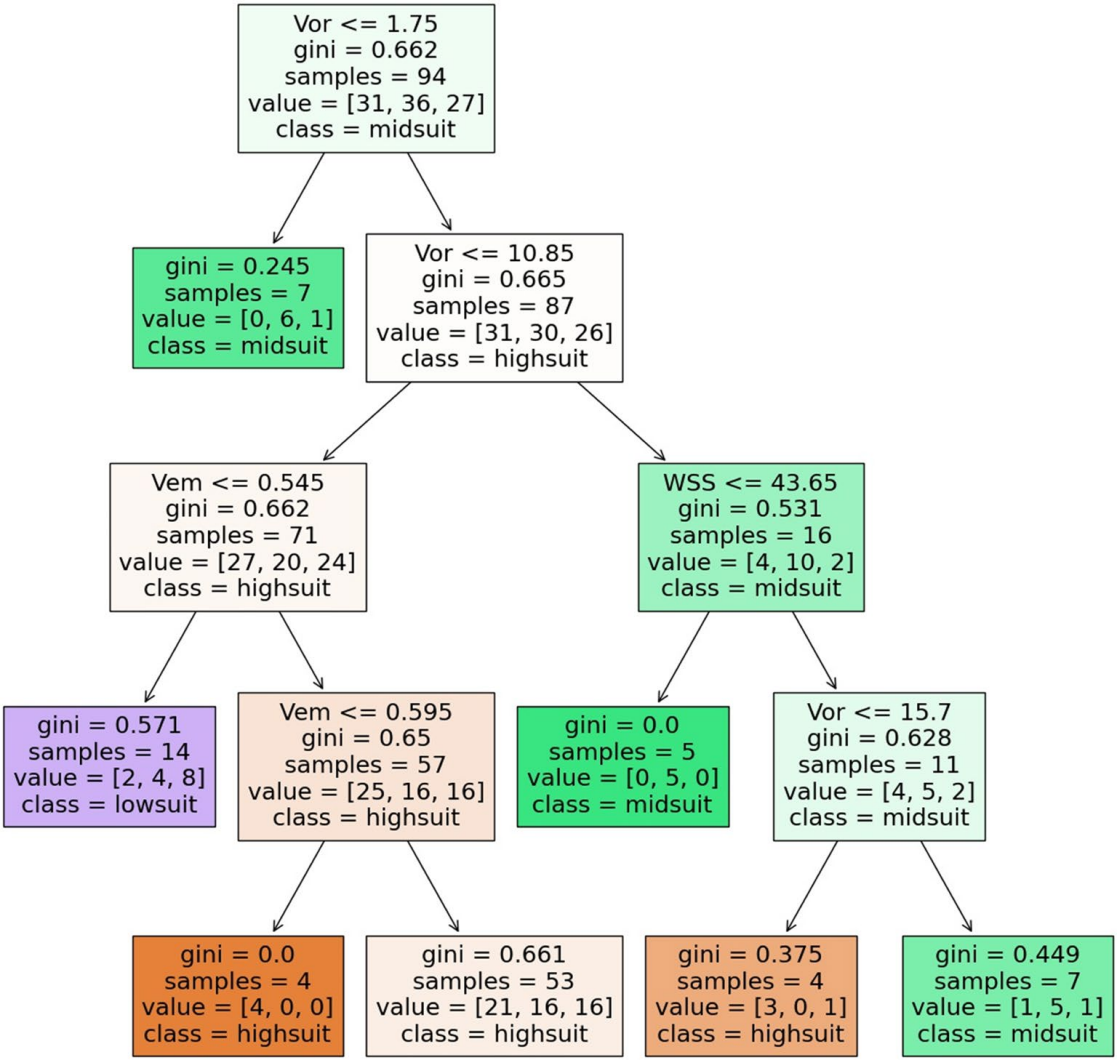
Decision tree for classification of the test fish built using CART.

Using the CART algorithm, the decision relationships between habitat suitability and flow velocity, vorticity, wall shear stress, and turbulent kinetic energy of the test fish were constructed. Based on the research results in Section 3.2, the model data set was composed of the habitat suitability of the test fish under 126 conditions and the corresponding 4 hydrodynamic indexes. The input eigenvalues were 4 hydrodynamic factors, and the output habitat suitability was divided into three categories: high suitability, medium suitability, and low suitability. During the monitoring period, the average occupancy rate per meter in the 140‐m river section should be approximately 100%/140 m, or about 0.7% per meter. Flow field environments with occupancy rates less than half this average (< 0.35%/m, corresponding to a habitat suitability index of 0.03) were classified as low suitability. Those exceeding twice the average (> 1.4%/m, corresponding to a habitat suitability index of 0.15) were classified as high suitability. Environments with occupancy rates between these thresholds (0.35%/m–1.4%/m, corresponding to a habitat suitability index of 0.03–0.15) were classified as medium suitability. The darker the color of the generated classification tree nodes, the lower the Gini coefficient and the better the classification effect of the model. According to the decision trees generated from the training set, the hydrodynamic factor requirements for different suitability flow field environments were derived, and thus, the suitable hydrodynamic factor selection mechanisms for test fish habitats were derived (Figure 6, Table 2).

**TABLE 2:** Hydrodynamic selection mechanism of suitable habitat for test fish.

*Onychostoma sima*			
Number	Selection mechanism	Suitability	Accuracy
1	10.85/s < Vor ≤ 15.7/s, WSS > 43.65 Pa	High‐suit	100%
2	1.75/s < Vor ≤ 10.85/s, Vem > 0.545 m/s	High‐suit	43.90%
3	Vor ≤ 1.75/s	Mid‐suit	85.70%
4	Vor > 15.7/s, WSS > 43.65 Pa	Mid‐suit	71.40%
5	Vor ≥ 10.85/s, WSS ≤ 43.65 Pa	Mid‐suit	100%
6	1.75/s < Vor ≤ 10.85/s, Vem ≤ 0.545 m/s	Low‐suit	57.10%

The vorticity of highly suitable habitat for *O. sima* was mostly between 1.75 and 10.85/s, and high flow velocity (> 0.545 m/s) or high shear stress of the bed body (> 43.65 m^2^/s^2^) should exist at the same time while meeting this condition. If the flow velocity in this vorticity interval is too low (≤ 0.545 m/s), there is a 57.1% possibility that this flow is not suitable for the habitat of *O. sima* .

**FIGURE 7:**
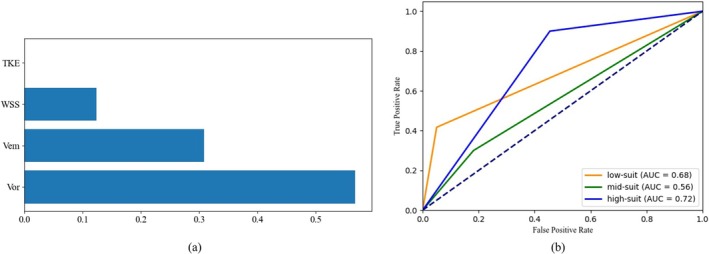
Ranking the importance of hydrodynamic factors on habitat suitability (a). ROC curves for predicting habitat suitability based on hydrodynamic selection mechanisms (b).

The results of decision tree generation showed that velocity was the most important hydrodynamic factor affecting the habitat suitability of *O. sima* , indicating that different hydrodynamic factors have different degrees of influence on the habitat of fish (Figure 7a). The data from the validation set were loaded into the model, and the selection mechanism of hydrodynamic factors suitable for the test fish habitat was verified by the ROC curve (Figure 7b). The results showed that the model predicted the suitable habitat of *O. sima* with a high success rate, and the AUC values of the ROC curves were 0.68, 0.56 and 0.72 for the low, medium and high suitability habitats.

**FIGURE 8:**
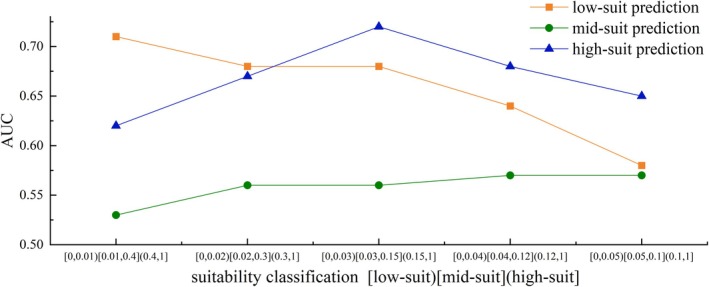
Analysis of model predictive accuracy.

To validate the accuracy of the suitability classification method employed in this study, five habitat suitability index classification schemes were established based on the average occupancy rate as the central reference, with the suitability intervals defined as follows: Interval 1 ([0, 0.01), [0.01, 0.4), (0.4, 1]); Interval 2 ([0, 0.02), [0.02, 0.3), (0.3, 1]); Interval 3 ([0, 0.03), [0.03, 0.15), (0.15, 1]); Interval 4 ([0, 0.04), [0.04, 0.12), (0.12, 1]); and Interval 5 ([0, 0.05), [0.05, 0.1), (0.1, 1]). Five classification decision trees were constructed using the CART method, and the mean AUC of each interval was calculated using the ROC method based on 10 randomized data splits (Figure 8). The results showed that Intervals 1 to 3 performed well in identifying low suitability zones, while none of the intervals achieved good classification results for medium suitability. Interval 3 showed the highest AUC for high suitability classification. Overall, comparative analysis indicates that the suitability thresholds defined in Interval 3 are the most appropriate for identifying habitat preference patterns of the target fish species in this study.

## 4 Discussion

### 4.1 Analysis of Suitable Habitat for Test Fish

The real‐time monitoring results of the study revealed that the complex flow field conditions shaped by microtopographic features exert varying degrees of attraction on the habitat of the test fish under different flow conditions. Moreover, within the same topography, different flow regions exhibit distinct levels of attraction at varying flow rates. The study assessed the habitat preference of the test fish under different flow field conditions based on six flow scenarios and 21 experimental zones for the test fish. Preliminary calibration of the preferred ranges for flow velocity, vorticity, bed shear stress, and turbulent kinetic energy associated with the preferred habitat of the test fish was conducted. Furthermore, we explored the habitat selection mechanisms of the test fish based on multiple hydrodynamic indicators. The following conclusions were drawn: Flow velocity and vorticity are important indicators influencing the habitat preference of *O. sima* . In the experimental environment, *O. sima* did not exhibit a fixed preference for highly suitable habitats. The preferred habitats with high vorticity typically fell within the range of 1.75 to 10.85/s, while also requiring high flow velocity (> 0.545 m/s) or high bed shear stress (> 43.65 m^2^/s^2^). If the flow velocity within this vorticity range was too low (≤ 0.545 m/s), the likelihood of low‐suitability habitats increased.

### 4.2 Rationality and Examinability of the Study

*O. sima* prefers to live in water environments with gravelly substrates and more turbulent currents (Mo et al. 2014). The experimental results indicate that none of the test fish were impacted by the flow in the barrier net under high‐velocity conditions in the test reaches with sand and gravel substrates, and there was a significant habitat preference for some of the high‐velocity areas. The test results did not conflict with the historical data. Some scholars conducted studies on the swimming ability of the test fish. The response flow speed of *O. sima* was between 0.4 and 1.0 BL/s; the critical swimming speed is between 5.8 and 9.2 BL/s, and the burst swimming speed is between 7.4 and 9.9 BL/s (Ding et al. 2020). Flow velocities throughout the test area under all conditions were within the appropriate flow velocity range for the test fish, except for some of the conditions in the branching section of the sandbar reach (almost no test fish inhabited for a long time).

**FIGURE 9:**
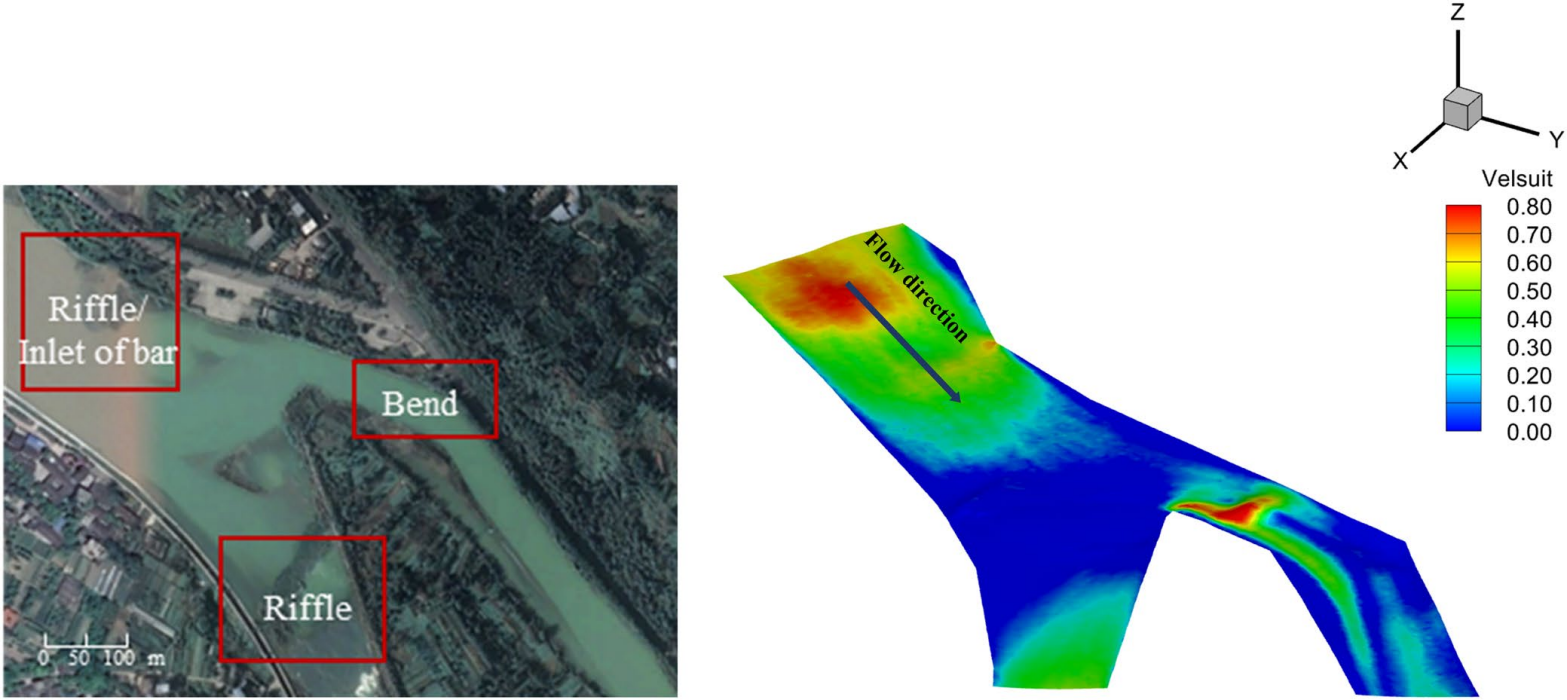
Distribution of flow field in typical spawning field.

Based on the survey results of the impact area of the Angu Hydropower Station, the left river network of Angu Hydropower Station maintains a constant ecological discharge flow of 100 m^3^/s. The Fengdumiao spawning ground is a typical spawning site for *O. sima* in the region. A three‐dimensional analysis of the hydrodynamic conditions at this natural spawning site reveals that the bottom layer contained several high‐flow velocity environments highly suitable for *O. sima* , consistent with the experimental findings (Figure 9). These highly suitable areas are located at river bends or riffles, where vortices of certain sizes are present. The actual conditions align with the experimental results, suggesting that the experimental findings have practical application prospects.

### 4.3 The Application Prospect of the Research

Restoration of fish habitat is the focus of current river ecological restoration work. Various ecological water conservancy projects have been carried out all over the world, such as the construction of artificial weirs (such as V‐weirs and W‐weirs) in riverbeds and the placement of fallen logs in rivers (Feld et al. 2011). The hydraulic conditions of each restoration project have been studied at the laboratory scale, but the laboratory conditions are limited by flow rate, substrate conditions, light conditions, fish activity range, and other features, making it difficult to apply them directly to actual engineering construction. Meanwhile, rheophilic fish‐dominated populations exhibit significant environmental dependency, and the effectiveness of existing ecological restoration measures in most rivers around the world remains unclear. Monitoring of two restored rivers in northwestern Indiana (Juday Creek and Potato Creek) employing measures such as meander restoration revealed that most distribution metrics for rheophilic fish did not exceed pre‐restoration levels in the reconstructed reaches. Furthermore, the American brook lamprey ( *Lampetra appendix* ) was not collected post‐restoration due to geomorphic alterations and reduced flow velocity in restored sections (Moerke and Lamberti 2003). The Danube streber ( *Zingel streber* ), which relies on high flow rates (> 0.3 m/s), has suffered from reduced flow rates, habitat loss, and population isolation due to dam construction. Its secretive nocturnal behavior has contributed to the lack of ecological data for this data‐deficient fish (Brinker et al. 2018; Fruget et al. 1998). Studies of 18 rehabilitated reaches along the lower River Rhine demonstrated the critical importance of habitat heterogeneity and connectivity for riverine fish biodiversity, indicating that tailored restoration approaches are required for different fish guilds.

Determining how to clarify the internal mechanism of the difference in the project restoration effect and how to transform the existing river structural restoration project to achieve the designed effect of attracting fish habitat are the focus of current river restoration work. The successful reproduction of rheophilic species in natural spawning areas alone does not reliably predict subsequent population development (Knott et al. 2021). Research across 13 river reaches located in the Austrian provinces of Upper Austria, Lower Austria, Styria, and Carinthia advocates integrating mesohabitat characteristics with rheophilic fish requirements to inform river management (Hauer et al. 2011). Similarly, Japan and China have established mesoscale experimental facilities simulating natural habitats for fish preference studies (Liu et al. 2023; Mochizuki et al. 2006). This study quantifies the relationship between the hydrodynamic demand and fish habitat preference of medium‐ and small‐scale river ecological restoration projects based on near‐natural rivers, providing a theoretical basis and technical support for the improvement of river structural restoration project effects.

### 4.4 Limitations of the Study and Methods for Future Improvement

The experimental precision needs to be further improved. The PIT method is limited by signal interference, which necessitates restricting the number of monitoring coils, thereby reducing data density. The behavioral trajectory of test fish can be accurately measured by acoustic methods on a larger scale to reduce the amount of data loss. However, at the same time, there are also adverse effects, such as an increase in monitoring cost, a decrease in test samples, and an increase in external influencing factors. The spatial resolution of radio telemetry is insufficient to meet the experimental requirements at the scale of localized microtopography. Different monitoring methods can be tested for suitability within the experimental area.

The range of fish species and age should be expanded. The Dadu River, where the study area is located, is home to several rare and valuable fish species, including *Leptobotia elongata* , *Myxocyprinus asiaticus* , and *Rhinogobio ventralis* . The selection of more types of local typical fish and the selection of brood fish/juvenile fish at different ages can provide more detailed hydraulic requirements for the construction of river ecological restoration projects. Concurrently, the test fish used in the experiment were artificially cultured species. Cognitive abilities and behavioral patterns differed between these cultured fish and wild conspecifics (Svitacova et al. 2025). Despite undergoing an acclimatization period to semi‐natural flow conditions in experimental channels, results may deviate significantly from those observed in natural environments.

Most fish species, particularly migratory taxa, exhibit distinct habitat shifts across life stages (Harwood and Babaluk 2014; Kargenberg et al. 2020). During reproduction, they target optimal spawning and incubation conditions; in foraging phases, they maximize energy acquisition efficiency by selecting prey‐rich river segments; while wintering prioritizes survival assurance. Consequently, experimental designs need to encompass all critical life stages to accurately quantify habitat requirements. Fish monitoring in a single trial can be extended from a single day to multiple days to enhance the reliability of the results. According to the main habitat reaches of fish in different periods, different hydrodynamic and topographic requirements can be determined, and appropriate habitat modification measures can be implemented in the spawning ground, foraging ground, and wintering ground of the target fish.

## 5 Conclusion

In this study, we selected the typical economic and protected fish species, *O. sima* , in the complex river network area of the test site. Based on the ecological test site with near natural conditions, various microterrains, such as straight lines, curved lines, sandbars, pools, and riffles, were shaped, and RFID technology was used to monitor the habitat behavior preferences of the test fish under multiterrain conditions in real time. Four indexes, velocity, vorticity, turbulent kinetic energy, and wall shear stress, were selected to explore the deep hydrodynamic mechanism of different suitable habitats for fish and the habitat behavior selection mechanism of fish under complex flow field conditions.

The following conclusions were drawn: (1) The complex flow field conditions shaped by microtopographic features exert varying degrees of attraction on the habitat of the test fish under different flow conditions. In the experimental environment, as the flow rate increased, *O. sima* 's preferred habitat gradually shifted from straight river segments to more complex terrain segments such as pool‐riffle and sandbar segments. Specifically, in the pool‐riffle segments, the fish predominantly preferred to reside around the riffles, while in the sandbar segments, they were mostly found near the inlets and outlets of the sandbars. (2) Vorticity and flow velocity are the primary hydrodynamic indicators influencing the habitat preference of *O. sima* . The flow conditions of the highly suitable habitats for *O. sima* are typically characterized by vorticity values ranging between 1.75 and 10.85/s, with flow velocities exceeding 0.545 m/s. (3) The study provides hydrodynamic threshold indicators that can be applied to ecological restoration practices, based on large‐scale ecological experiments addressing the actual habitat needs of fish. These indicators offer a theoretical basis that can be extended to practical applications such as the shaping of microtopography for fish habitats and the identification of potential fish habitats.

## Author Contributions

**Han Liu:** conceptualization (lead), data curation (equal), formal analysis (equal), writing – original draft (lead), writing – review and editing (equal). **Guosheng Yang:** writing – review and editing (equal), methodology (equal). **Junqiang Lin:** conceptualization (equal), investigation (supporting), methodology (equal). **Dongsheng Wang:** investigation (supporting), project administration (supporting), resources (equal), supervision (equal). **Hao Jiang:** methodology (equal), project administration (equal), resources (equal). **Wei Xu:** data curation (supporting), methodology (supporting). **Di Zhang:** data curation (supporting), formal analysis (supporting). **Lingquan Dai:** investigation (supporting), methodology (supporting). **Wei Jiang:** project administration (supporting), validation (equal), visualization (equal). **Sha Li:** project administration (supporting), validation (supporting), visualization (supporting).

## Ethics Statement

This study was performed in line with the principles of the Declaration of Helsinki and the guidelines and regulations of the National Institute of Health Guide for the Care and Use of Laboratory Animals, China. We received permits to conduct this research from the ethics board of Chinese Sturgeon Research Institute, Yichang, China (Approval ID: CSRI2021625; Approval Date: June 27, 2021).

## Conflicts of Interest

The authors declare no conflicts of interest.

## Data Availability

The authors declare that the data supporting the findings of this study are available within the paper. The data that support the findings of this study are available in Dryad 10.5061/dryad.05qfttfcx. Should any raw data files be needed in another format, they are available from the corresponding author upon reasonable request.
